# Pregnancy exposure of titanium dioxide nanoparticles causes intestinal dysbiosis and neurobehavioral impairments that are not significant postnatally but emerge in adulthood of offspring

**DOI:** 10.1186/s12951-021-00967-5

**Published:** 2021-08-06

**Authors:** Junhao Su, Xinhao Duan, Yu Qiu, Lixiao Zhou, Hongyang Zhang, Min Gao, Yijun Liu, Zhen Zou, Jingfu Qiu, Chengzhi Chen

**Affiliations:** 1 Department of Health Laboratory Technology, School of Public Health and Management, Chongqing Medical University, Chongqing, 400016 People’s Republic of China; 2 Department of Neurology, The Affiliated University-Town Hospital of Chongqing Medical University, Chongqing, 401331 People’s Republic of China; 3 Molecular Biology Laboratory of Respiratory Diseases, Institute of Life Sciences, Chongqing Medical University, Chongqing, 400016 People’s Republic of China; 4 Dongsheng Lung-Brain Disease Joint Lab, Chongqing Medical University, Chongqing, 400016 People’s Republic of China; 5 Department of Occupational and Environmental Health, School of Public Health and Management, Chongqing Medical University, Chongqing, 400016 People’s Republic of China

**Keywords:** Titanium dioxide nanoparticles, Intestinal dysbiosis, Neurobehavioral impairments, Pregnancy exposure

## Abstract

**Background:**

Pregnancy exposure to titanium dioxide nanoparticles (TiO_2_NPs) is a vital consideration due to their inadvertent ingestion from environmental contamination. The potential health effects of TiO_2_NPs on the neurodevelopmental process should be seriously concerned in health risk assessment, especially for the pregnant women who are susceptible to the neurodevelopmental toxicity of nano-sized particles. However, the available evidence of neurodevelopmental toxicity of TiO_2_NPs remains very limited.

**Methods:**

In the present study, the pregnant mice were intragastric administered with 150 mg/kg TiO_2_NPs from gestational day (GD) 8 to 21, the maternal behaviors and neurodevelopment-related indicators in offspring were all assessed at different time points after delivery. The gut microbial community in both dams and their offspring were detected by using 16S ribosomal RNA (rRNA) gene sequencing. The gut-brain axis related indicators were also determined in the offspring.

**Results:**

The results clearly demonstrated that exposure to TiO_2_NPs did not affect the maternal behaviors of pregnant mice, or cause the deficits on the developmental milestones and perturbations in the early postnatal development of offspring. Intriguingly, our data revealed that pregnancy exposure of TiO_2_NPs did not affect locomotor function, learning and memory ability and anxiety-like behavior in offspring at postnatal day (PD) 21, but resulted in obvious impairments on these neurobehaviors at PD49. Similar phenomena were obtained in the composition of gut microbial community, intestinal and brain pathological damage in offspring in adulthood. Moreover, the intestinal dysbiosis induced by TiO_2_NPs might be highly associated with the delayed appearance of neurobehavioral impairments in offspring, possibly occurring through disruption of gut-brain axis.

**Conclusions:**

This is the first report elucidated that pregnancy exposure to TiO_2_NPs caused delayed appearance of neurobehavioral impairments in offspring when they reached adulthood, although these perturbations did not happen at early life after delivery. These findings will provide valuable insights about neurodevelopmental toxicity of TiO_2_NPs, and call for comprehensive health risk assessment of TiO_2_NPs on the susceptible population, such as pregnant women.

**Graphical abstract:**

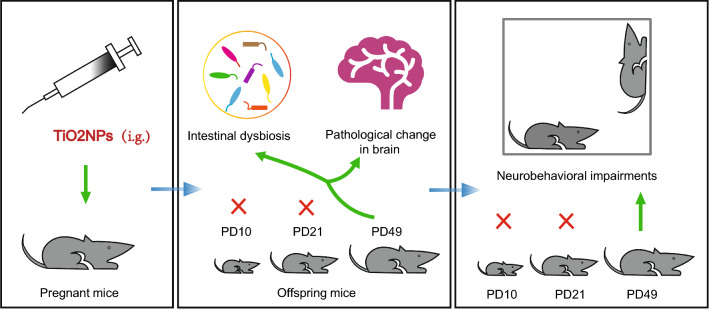

**Supplementary Information:**

The online version contains supplementary material available at 10.1186/s12951-021-00967-5.

## Introduction

Titanium dioxide (TiO_2_) is one of the most renowned transition metal oxides in the environment. It has been widely applied in many commercial and industrial products, such as paints, coatings, plastics, cosmetic products, and food additives etc. [1, 2]. Approximately 8.4 million tons of pigmentary TiO_2_ is consumed annually on the global scale in 2020, and is expected to increase exponentially in the coming decades [3]. Notably, abundant applications of TiO_2_ are benefit from the small particle sizes, and thus, TiO_2_ is commonly produced in or near the nano range [1, 4]. The nanoscale TiO_2_ in the foods or consumer products can be excreted in the feces/urine, or disposed as wastes that inevitably enters the ecosystem [5, 6]. Consequentially, the majority of nanoscale and larger-sized TiO_2_ are found in influent sewage, and even the river water [7, 8]. Therefore, the increasing production of nano-sized TiO_2_ has received growing concerns about the consequences of human exposure through direct contact in the environment.

Traditionally, TiO_2_ nanoparticles (TiO_2_NPs) are considered as poor soluble metal oxide nanoparticles with relatively low acute or chronic toxicity [9]. However, this view is now challenged in recent years due to increasing evidence has revealed that TiO_2_NPs are capable of entering human body and inducing pathological damage in various organs, such as liver, kidney and brain etc. [10–12]. Furthermore, different animal models and cultured cell lines are intensively applied for safety assessment of TiO_2_NPs employing multiple exposure ways of treatment, including inhalation, ingestion and intraperitoneal injection [9, 13]. These investigations also confirmed the notion that, TiO_2_NPs might present a high risk to human health when exposed in the daily life, particularly in the sensitive period of lifespan, such pregnancy [14, 15]. Of note, previous studies have revealed that, during the embryonic stage, maternal inhaled nanoparticles can cross the placental barrier into the embryonic blood circulation system, and therefore, affecting the entire embryonic development or causing direct adverse consequences in the developing offspring [16]. Indeed, transport of TiO_2_NPs in systemic circulation and further transition through placental barrier are well documented [15, 17–19]. From the developmental point of view, exposure to TiO_2_NPs during critical windows at pregnancy period therefore received a raising concern. In fact, in either fertile age or embryonic period, exposure of TiO_2_NPs in human mothers, their children are very likely to be exposed to high cumulative doses [15, 17]. By using animal-exposed models, several toxicological studies have correspondingly demonstrated that ingestion of TiO_2_NPs during pregnancy results in diverse brain dysfunction of offspring, such as learning and memory impairments [18] and behavioral deficits relevant to autism spectrum disorder [19]. However, to date, the underlying neurodevelopmental toxic mechanisms of TiO_2_NPs are still poorly understood.

Gut microbiota is largely responsible for human health through a wide range of biological and metabolic activities [20]. Although the microbial composition of adult is relatively stable, it is well established that the diversity of microbiota is acquired very early in life after delivery and can thereafter be shaped over time [21]. The normal gut microbiota is usually reshaped by the several major factors, including genotype, mode of delivery, diet composition during infancy and adulthood, use of drugs and environmental exposure to xenobiotics [21, 22]. Notably, among these influence factors, mounting evidence has now revealed that disruption of the normal microbial community is a known deleterious effect of environmental xenobiotics. The biotransformation of xenobiotics may in turn be essentially affected by the gut microbiota [21–23]. Likewise, the toxic endpoints regarding the adverse effects of xenobiotics on the intestinal microbiota are included in many toxicity test systems [22, 23]. Intriguingly, environmental exposures during pregnancy are also able to disrupt the gut microbiome of offspring [24, 25], which may further lead to unpredictable impact on their postnatal development. Nevertheless, the role of gut microbiota in the pregnancy exposed TiO_2_NPs-induced neurodevelopmental toxicity remains largely unknown.

Therefore, in this study, the pregnant animal model was used to explore whether exposure to TiO_2_NPs during critical period of embryonic development could cause abnormal changes in the major neurobehaviors of offspring. To further elucidate the role of intestinal microbiota involved in this process, the fecal samples of dams and offspring were accordingly collected at different time points after birth and subjected to the 16S ribosomal RNA (rRNA) sequencing. Interestingly, the results demonstrated for the first time that pregnancy exposure of TiO_2_NPs resulted in dysbiosis and neurobehavioral deficits that were not significant postnatally but emerged in adulthood of offspring. These findings may offer a novel insight regarding mechanistic basis about neurodevelopmental toxicity of TiO_2_NPs, and call for comprehensive risk assessment on the toxic response of gut microbiome.

## Materials and methods

### Animals and treatment

**Fig. 1:**
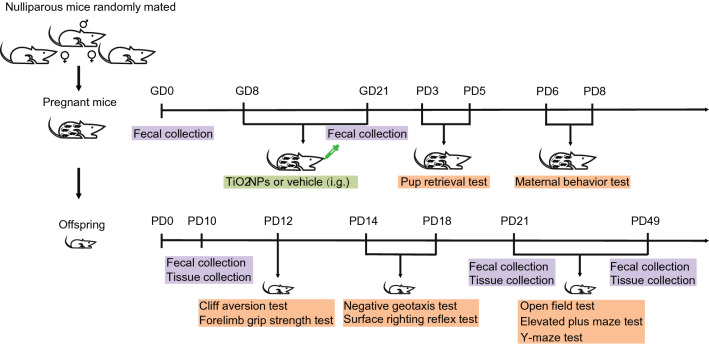
The experimental design of this study

Eight-week-old healthy C57BL/6 J mice, weighted 20–24 g, were obtained from Hunan Silaike Jingda Laboratory Animal Co., Ltd. (Hunan, China). They were kept in the Experimental Animal Center of Chongqing Medical University under a controlled condition (temperature: 23 ± 1 °C, relative humidity: 50 ± 10% and a 12 h/12 h light/dark cycle). All the mice were free access to rodent food and tap water. After adapting to varying environmental conditions for 1 week, the unmated female and male mice were mated overnight at the ratio of 2:1. The vaginal plug was checked at 8:00 A.M. in the next morning. The day when the copulatory plugs occurred was regarded as the gestational day 1 (GD1). A total of forty pregnant animals were randomly divided into the vehicle group and TiO_2_NPs treatment group using a randomization tool on the website www.randomizer.org. In each group, 20 pregnant mice were intragastric exposed with either vehicle or 150 mg/kg TiO_2_NPs suspension solution from GD8 to GD21. The intragastric dosage of TiO_2_NPs was chosen and calculated based on Chinese Standard for Food Additives (GB2760-2015). The animals were administrated with TiO_2_NPs once a day between 9:30 AM and 10:30 AM to avoid the potential influence of circadian rhythm. The delivery day was considered postnatal day 0 (PD0). After administration of TiO_2_NPs, the pregnant mice were permitted to deliver spontaneously. The offspring were weaned at PD21 and thereafter isolated. The birth of pregnant mice were checked twice a day in the morning and evening. After birth, five female pups and five male pups were randomly assigned to each group for the designed experiments, and there could be no more than 1 female and 1 male from the same litter. The experimental design was shown in Fig. 1. Sufficient efforts were made to minimize the needless suffering of laboratory animals. All the animal experiments were approved by the Animal Administration and Ethics Committee of Chongqing Medical University, and were carried out according to the guideline.

### Preparation and characteristics of TiO_2_NPs

**Fig. 2:**
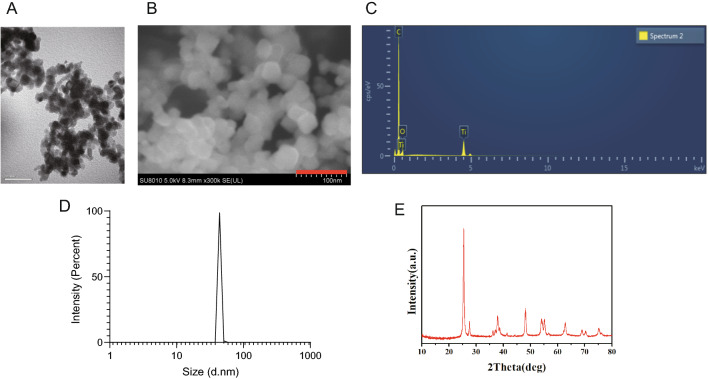
Characteristics of TiO_2_NPs used in this study. **A** Transmission electron microscope image of TiO_2_NPs. **B** Scanning electron microscopy image of TiO_2_NPs. **C** Chemical composition of TiO_2_NPs. **D** The hydrodynamic diameter of TiO_2_NPs was determined by dynamic light scattering. **E** The crystal structure of TiO_2_NPs was detected by X-ray diffraction

TiO_2_NPs nano-powder, 21 nm particle size, surface area 35–65 m^2^/g, density 4.26 g/mL at 25 °C, were obtained from Sigma Aldrich Chemical Co. (Cat: 718467, MO, USA). At first, the nano-powder were diluted in heat-inactivated serum in MilliQ water. Subsequently, the solution was sonicated with an ultrasonic cleaner (SB-5200DT, Ningbo Scientz Biotechnology Co., Ltd, Ningbo, China) at 20% of maximum amplitude for 20 min on ice. The suspended solution of TiO_2_NPs was freshly prepared before use, and the vehicle control was used by sonication of 2% heat-inactivated serum in MilliQ water. The particles in the solution were then observed by transmission electron microscopy (Hitachi-7500; Hitachi, Ltd, Tokyo, Japan). Field emission scanning electron microscopy (Hitachi SU8010) with energy dispersive spectroscopy (Oxford X-MAN 50) (FE-SEM/EDS) was used to analysis the chemical elemental composition. The hydrodynamic diameter of TiO_2_NPs was determined by dynamic light scattering (DLS) using Malvern Zetasizer Nano ZSP (Malvern, Massachusetts, USA). The crystal structure of TiO_2_NPs was assessed by X-ray diffraction (XRD) using X'Pert Pro MPD (PANalytical, Almelo, Netherlands). All the  characteristics of TiO_2_NPs was shown in Fig. 2.

### Maternal behavior tests

#### Maternal behavior assessment

Maternal behaviors were assessed based on the protocols reported previously [26, 27]. The frequencies of eating, drinking, self-grooming or licking, pups licking and nest building, were all recorded from 9:00 A.M. to 11:00 A.M at PD6 and PD8 by two independent observers who were blinded to the treatment of animals. Each dam was continuously monitored by the same designated observer. The frequency of each behavior was recorded during 15 min observation period.

#### Pup retrieval test

Pup retrieval test was carried out as described previously [28]. On PD3 and PD5, the latency to retrieve each pup back into the nest was assessed, respectively. The dams were separated from their pups before test. Then, the pups in each litter were scattered to four corners of the cage, and the latency of dam that collected the pups back in the nest was assessed for 15 min.

### Developmental milestones assessment

The appearance of developmental milestones, including development of fur, incisor eruption, eye opening, vaginal opening and testes descent were observed daily between 9:00 A.M. to 11:00 A.M until each milestone attained. The definition of fur development was that the whole body seemed to be covered with fur. Incisor eruption referred to the first time the upper incisor appeared. Eye opening of animals were the complete separation of double eyelids. Vaginal opening was the opening of the vaginal cavity to the skin, and testes descent referred to the testis began to descend into the scrotum. In addition, all the body weight of offspring were recorded from PD1 to PD49.

### Neonatal sensory and motor development assessment

#### Cliff aversion test

Cliff aversion test was conducted based on the methods reported previously [28]. Briefly, the animals were gently placed on the edge of a platform, with their front paws contacting and facing the edge of platform. The latency of mouse crawled away from the edge was measured. Each pup was tested between 9:00 A.M. and 11:00 A.M. at PD12.

#### Forelimb grip strength test

Forelimb grip strength test was performed based on the protocols reported previously [28]. In brief, the forelimbs of animal were placed on a thin iron wire. The time that for the pup grasped on the wire was measured. Each pup was tested between 9:00 A.M. and 11:00 A.M. at PD12.

#### Surface righting reflex test

Surface righting reflex test was carried out based on the method reported previously [28]. In short, the mouse was gently placed in a supine position on a warm rough horizontal plate. The latency of pup flipped by itself until all four feet touched the plate was recorded. The test was lasted for 120-s observation period. Each pup was tested for surface righting reflex between 9:00 A.M. and 11:00 A.M. on PD14 and PD18.

#### Negative geotaxis test

Negative geotaxis test was conducted to evaluate the motor coordination in offspring [28]. In brief, the mice were gently placed facing down on a 25-degree inclined plate. Then, the latency of animal that completed a 180-degree turn on the slope plate was recorded. The test lasted for 120-s observation period. Each mouse was assessed once every day between 9:00 A.M. and 11:00 A.M. at PD14 and PD18.

### Open-field test

The open-field test was conduct to evaluate the locomotor activity [29]. In short, the animals were placed initially in the central area of open-field apparatus facing the same direction for 5 min of testing. The total distance, central square duration and distance moved in center were recorded by the tracking system with video camera installed above the apparatus. The apparatus was also cleaned with 75% ethanol carefully between each trial. Each animal was tested once every day between 8:00 A.M. and 12:00 A.M. at PD21 and PD49.

### Elevated plus maze

Elevated plus maze test was conducted according to the procedures reported previously [28]. In brief, the animals were placed initially on the central platform of maze facing one of the closed arms for 5 min of testing. Number of entries into the opened arm, number of head-dipping in the opened arm, the open arms duration, and distance moved in the open arms were all recorded by the video camera tracking system. The apparatus was carefully cleaned between each trial, and each animal was tested once every day between 8:00 A.M. and 12:00 A.M. at PD21 and PD49, respectively.

### Y maze

#### Spontaneous alternation test

The spontaneous alternation test was performed based on the protocols reported previously [30]. Briefly, the animals were placed initially in the distal end of arm for 5 min of testing. The arm entries and the alternations (entry into an arm that differs from the previous two entries) were recorded by a tracking system with video camera installed above the apparatus. Subsequently, the percentage of alternations was calculated based on the following formula: (Alternations/Arm Entries-2) × 100%. The maze was carefully cleaned with 75% ethanol between each trial. Each animal was tested once every day between 8:00 A.M. and 12:00 A.M. at PD21 and PD49.

#### Novelty discrimination test

Y-maze novelty discrimination test was carried out based on the procedures reported previously [31]. The test consisted of a 5-min acclimatization phase and 5-min test phase, and the experimental interval between these two phases lasted for 1 h. The animals were placed in the distal end of the acclimatization arm with the novel arm closed. In the test phase, the mouse was allowed to explore both familiar and unfamiliar arms for 5 min. The novel arm duration, and distance moved in the novel arm were recorded by the tracking system with video camera installed above the apparatus. Each animal was tested once every day between 8:00 A.M. and 12:00 A.M. at PD21 and PD49.

### Hematoxylin–eosin staining

Hematoxylin–eosin staining was conducted based on the procedures reported previously [32]. Briefly, after the animals were sacrificed, the intestinal and brain tissues were quickly dissected on ice and fixed in fresh prepared 4% paraformaldehyde for morphological observation. The paraffin sections were dewaxed in xylene and treated with gradient doses of ethanol. After deparaffinization, the sections were stained with hematoxylin and eosin. The H&E stain kit was from Solarbio Science & Technology Co., Ltd. Beijing, China. The sections were subsequently dehydrated in ethanol, cleared in xylene, and mounted with neutral balsam. Lastly, the stained sections were observed under a light microscope.

### Alcian blue periodic acid schiff (AB-PAS) staining

AB-PAS staining was performed according to the procedures reported previously [33]. In brief, after the intestine tissues were fixed in fresh prepared 4% paraformaldehyde overnight, they were embedded in paraffin and cut into sections. Subsequently, the tissue sections were dewaxed with xylene, dehydrated in ethanol and stained using Alcian blue solution, Schiff reagent and hematoxylin, respectively. All stain solutions were from Solarbio Science & Technology Co., Ltd. Beijing, China. The sections were dehydrated using ethanol and cleared with xylene. Lastly, the sections were mounted using neutral balsam and observed under a light microscope.

### Toluidine blue staining

Toluidine blue staining was performed based on the protocols described previously [34]. In short, the intestine tissues were fixed in fresh prepared 4% paraformaldehyde and embedded in paraffin to cut into sections. Subsequently, the tissue sections were dewaxed with xylene, dehydrated in ethanol and stained by toluidine blue solution (Solarbio Science & Technology Co., Ltd. Beijing, China). After dehydration with ethanol, the sections were mounted using neutral balsam and observed under a light microscope.

### Immunofluorescence assay

Immunofluorescence assay was performed based on the methods described previously [32]. Briefly, the intestine tissues were quickly dissected on ice, and subjected to fixation in fresh prepared 4% paraformaldehyde. The frozen sections were prepared and washed with phosphate-buffered saline. After incubation with blocking solution for 1 h, the sections were further incubated with primary antibodies against TuJ1 (1:250, purchased from Abcam Co., Cambridge, UK) and HuC/D (1:250, obtained from Abcam Co., Cambridge, UK) at 4 °C overnight. On the next day, the sections were subsequently incubated with the fluorescent dye-conjugated secondary antibodies at room temperature for 1 h. Then, the nuclear of cell was visualized by 4’,6-Diamidino-2-phenylindole (DAPI). Lastly, the sections were observed under a fluorescence microscope.

### Quantitative PCR assay

Quantitative PCR assay was carried out based on the protocols reported previously [32]. Briefly, the total RNA was extracted from the intestine and brain tissues using TRizol reagent (KeyGen Biotech, Nanjing, China). The cDNA was generated using Perfect Real Time PrimeScript™ RT Master Mix. Real‐time quantitative PCR was conducted using TBGREEN Premix Ex Taq™ II (TliRNaseH Plus) on CFXConnect™ Real-Time System (Bio-Rad, Hercules, CA, USA). PCR reaction conditions were shown as follows: 95 °C for 2 min, followed by 40 amplification cycles of 95 °C for 5 s, 15 s at 60 °C and 20 s at 72 °C, then 65 °C and 95 °C for 5 s. All primers of target genes were synthesized by Sangon Biotech, Co., Ltd. (Shanghai, China, shown in Additional file 1: Table S1).

### Enzyme linked immunosorbent assay (ELISA)

The ELISA assay was conducted based on the procedures as previously described [32]. Mouse secretory immunoglobulin A (sIgA) and mouse diamine oxidase (DAO) ELISA kits were both from Cusabio Biotech Co. Ltd. (Wuhan, China). Briefly, the kits were used for determination after keeping at room temperature for at least 20 min. The samples were added into each well and incubated for 2 h. The biotin-antibody was then added and incubated for another 1 h at 37 °C. Next, horseradish peroxidase-conjugate was immediately added into the well followed by incubation for 1 h. Subsequently, the chromogenic substrate and stop solution were incubated in the dark, respectively. The absorbance was finally measured with a microplate reader at wavelength of 450 nm (Thermo Fisher Scientific Inc., Waltham, MA, USA).

### 16S ribosomal RNA gene sequencing

16S rRNA gene sequencing was conducted based on the procedures reported previously [32]. Briefly, after indicate treatment, the fecal samples of dams were collected under sterile conditions at GD0 and GD21, and the samples of offspring were collected at PD10, PD21 and PD49, respectively. The samples were all stored at −80 °C prior to use. After the total genomic DNA was extracted from samples, the bacterial 16S rRNA was amplified using the forward and reverse primers designed by adding a barcode to primers. The PCR amplification reaction was performed on the ABI GeneAmp 9700 (thermal cyclers from Applied Biosystems, CA, USA) using TransStart Fastpfu DNA polymerase. The PCR products were purified by AxyPrep DNA Gel Extraction Kit (Axygen Biosciences, CA, USA) and quantified by using QuantiFluor™-ST Blue Fluorescence Quantification System (Promega Co., WI, USA). MiSeq library was constructed for preparation of the fragment DNA, and the raw sequencing reads were obtained using Illumina MiSeq platform at Majorbio Bio Tech Co. Ltd (Shanghai, China).

### 16S ribosomal RNA gene sequencing analysis

The raw sequencing reads were clustered into operational taxonomic units (OTUs) based upon the similarity above 97% by using Majorbio’s cloud platform on the website https://cloud.majorbio.com (Majorbio, Shanghai, China). The Mothur software programs (version v.1.30.2) was applied to calculate the α-diversity of bacterial communities, including Shannon, Simpson, Chao, Ace and observed species. β-diversity was calculated by principal coordinates analysis (PCoA) in the R software.

### Statistical analysis

All obtained experimental data were reported as mean ± standard error of the mean (S.E.M.). The statistical analysis including one-way or repeated analysis of variance (ANOVA), independent student-*t* test and non-parametric Mann–Whitney U test were carried out by software of GraphPad Prism version 8.0 (GraphPad Software Inc., La Jolla, CA) when appropriate. *P* value less than 0.05 indicated statistical significance.

## Results

### Pregnancy exposure to TiO_2_NPs did not affect body weight, litter size and sex ratio of offspring

**Fig. 3:**
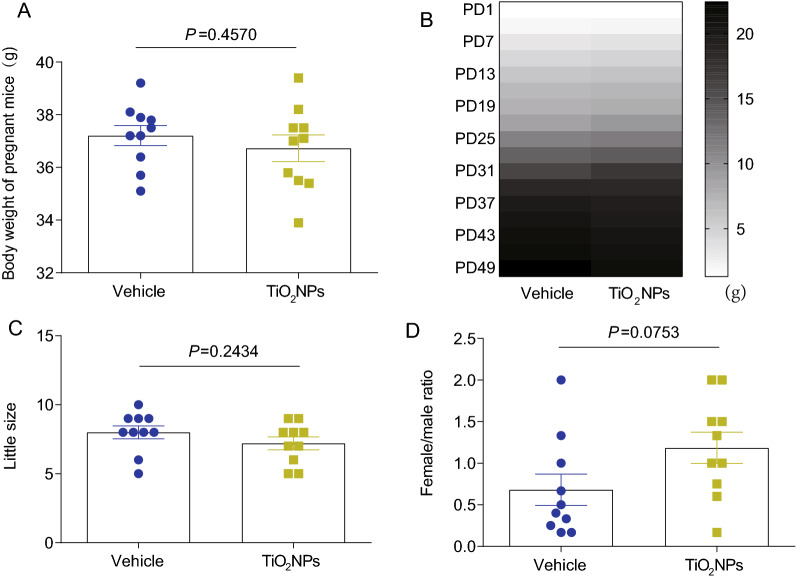
Pregnancy exposure to TiO_2_NPs did not affect body weight, litter size and sex ratio of offspring. **A** Effects of TiO_2_NPs on the body weight of pregnant mice. **B** Effects of TiO_2_NPs on the body weight of offspring from postnatal day (PD) 1 to PD49. **C** Effects of TiO_2_NPs on the little size. **D** Effects of TiO_2_NPs on the sex ratio (female/male) of offspring. Data were presented as mean ± S.E.M. Statistical analysis was performed by using independent student *t* test or Mann–Whitney U test

The influence of environmental xenobiotics on the body weight is usually assessed at the end of toxicological studies [35]. Thus, to evaluate the potential toxic effects of TiO_2_NPs during pregnancy exposure, the body weight was firstly measured. As shown in Fig. 3A, after treatment of TiO_2_NPs from GD8 to GD21, we did not find any significant changes on the body weight of pregnant mice between vehicle group and TiO_2_NPs-treated group. Similarly, no significant changes were obtained on the body weight of their offspring from PD1 to PD49 (Fig. 3B). The litter size and sex ratio of offspring also did not show any obvious alterations after exposure of TiO_2_NPs during pregnancy (Fig. 3C and D).

### Pregnancy exposure to TiO_2_NPs did not affect maternal behaviors of mice after delivery

**Fig. 4:**
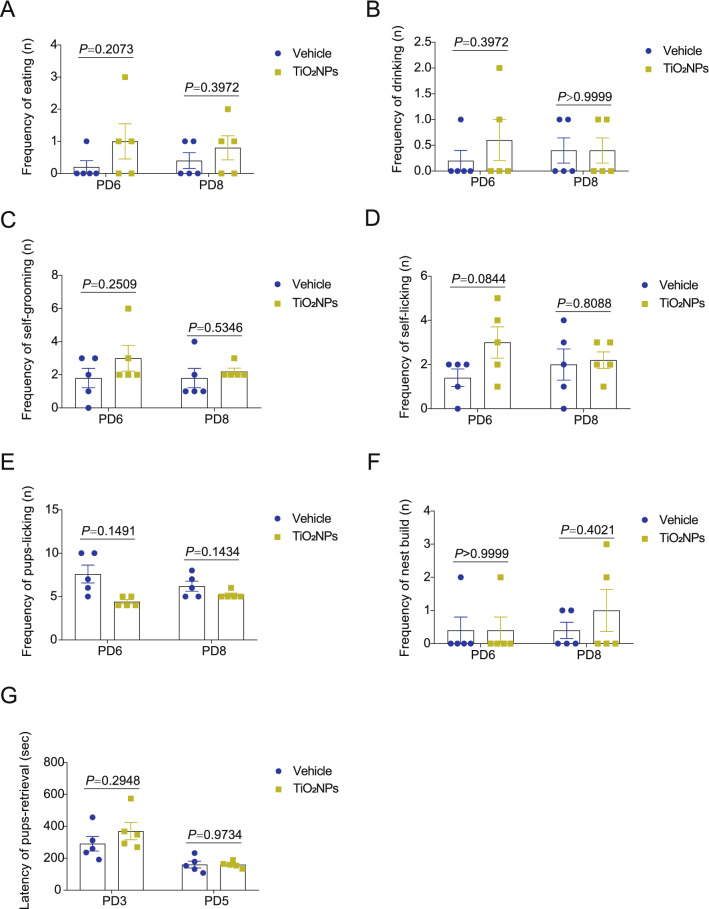
Pregnancy exposure to TiO_2_NPs did not affect maternal behaviors of mice after delivery. The maternal behaviors were assessed at postnatal day (PD) 6 and PD8. **A** Effects of TiO_2_NPs on the frequency of eating of dams. **B** Effects of TiO_2_NPs on the frequency of drinking of dams. **C** Effects of TiO_2_NPs on the frequency of self-grooming of dams. **D** Effects of TiO_2_NPs on the frequency of self-licking of dams. **E** Effects of TiO_2_NPs on the frequency of pups-licking. **F** Effects of TiO_2_NPs on the frequency of nest build. **G** The pup retrieval test was performed at PD3 and PD5, and the latency of pups-retrieval of each group was monitored. Data were presented as mean ± S.E.M. Statistical analysis was performed by using repeated-measure ANOVA or independent student *t* test or Mann–Whitney U test

Maternal care is a key determinant of offspring development. Additionally, the maternal care may facilitate the plasticity in offspring behaviors [36]. Thus, it is essential to test the potential impact on the maternal behaviors in response to environmental xenobiotics exposure during pregnancy. In this study, the data obtained from maternal behavioral observation showed that the frequency of eating, drinking, self-grooming, self-licking, pups-licking and nest build of dams did not alter obviously in TiO_2_NPs-treated group at PD6 and PD8 after delivery (Fig. 4A–F). Also, the result revealed that there was no apparent change on the latency of pups-retrieval between two groups at PD3 and PD5 (Fig. 4G). These results suggest that pregnancy exposure to TiO_2_NPs did not affect maternal behaviors of mice significantly.

### Pregnancy exposure to TiO_2_NPs did not cause the delays in developmental milestones and the perturbations in early postnatal development of offspring

**Fig. 5:**
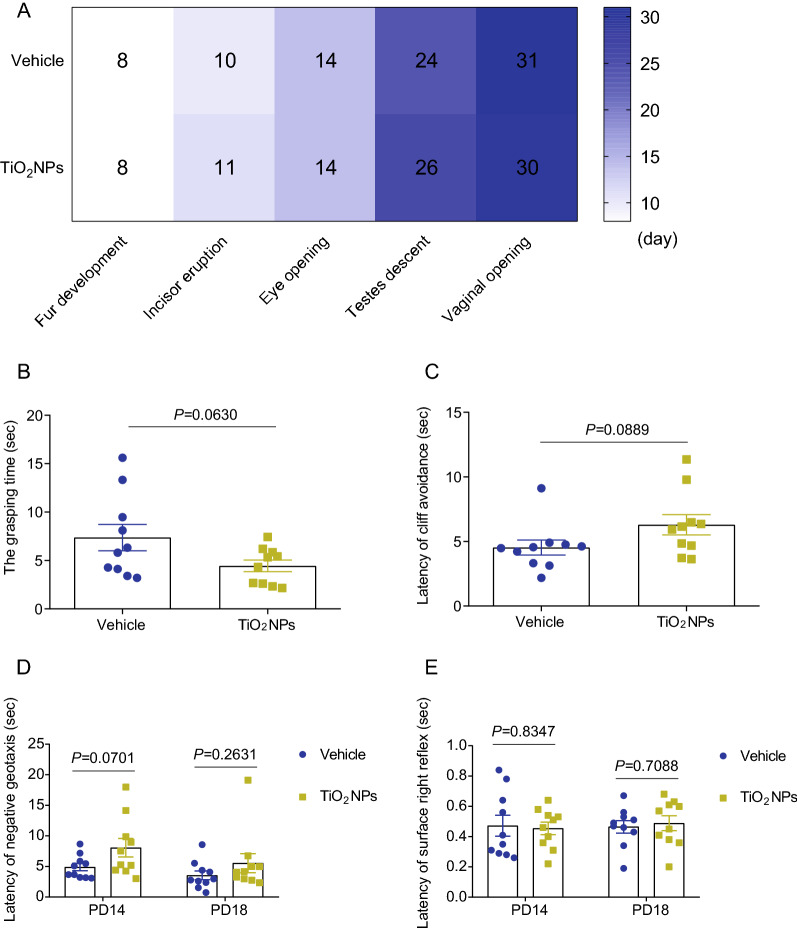
Pregnancy exposure to TiO_2_NPs did not cause the delays in developmental milestones and the perturbations in neurobehavioral development of offspring. The potential effects of TiO_2_NPs on the developmental milestones were assessed each day until each milestone attained. **A** Effects of TiO_2_NPs on the day of fur development, incisor eruption, eye opening, testes descent and vaginal opening in offspring. **B** Effects of TiO_2_NPs on the grasping time in the forelimb grip strength test at postnatal day (PD) 12. **C** Effects of TiO_2_NPs on the latency of cliff avoidance at PD12. **D** Effects of TiO_2_NPs on the latency of negative geotaxis at PD14 and PD18. **E** Effects of TiO_2_NPs on the latency of surface right reflex at PD14 and PD18. Data were presented as mean ± S.E.M. Statistical analysis was performed by using repeated-measure ANOVA or independent student *t* test or Mann–Whitney U test

Appearance of developmental milestones, including incisor eruption, development of fur, eye opening, testes descent and vaginal opening, were all assessed to evaluate the potential developmental toxic effects of TiO_2_NPs in offspring. As depicted in Fig. 5A, the preweaning development observations exhibited that there were no significant differences on these indicators between two groups. Similar phenomenon was observed on the reproduction development, showing no remarkable changes on both the appearance of testes descent and vaginal opening in offspring of TiO_2_NPs-treated animals as compared with vehicle controls. Although the grasping time of TiO_2_NPs-treated group seemed lower than that in vehicle group, this trend did not reach the statistical difference (Fig. 5B). Likewise, the latencies of cliff avoidance and negative geotaxis in the TiO_2_NPs-exposed animals were both slightly increased when compared with vehicle controls, but no significant differences were observed between two groups (Fig. 5C and D). The latency of surface right reflex in the TiO_2_NPs-treated group also did not change profoundly at PD14 and PD18 in comparison to control group (Fig. 5E). Taken together, these results indicate that pregnancy exposure to TiO_2_NPs may not cause the obvious perturbations in the early postnatal development of offspring.

### Pregnancy exposure to TiO_2_NPs inhibited the locomotor activity of offspring at PD49 but not occurred at PD21

**Fig. 6:**
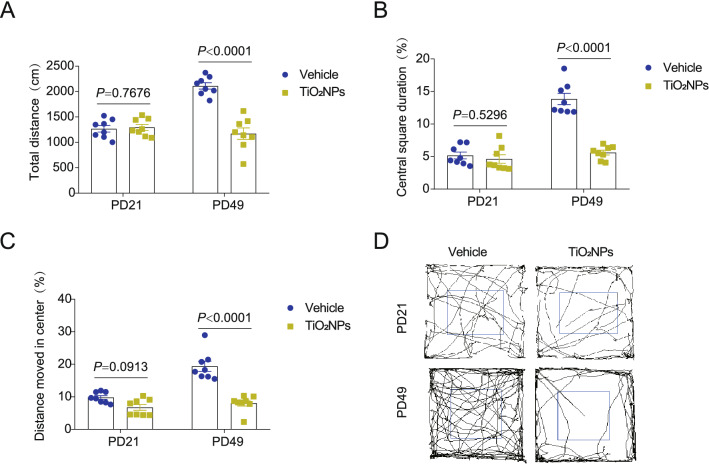
Pregnancy exposure to TiO_2_NPs inhibited the locomotor activity of offspring at PD49 but not occurred at PD21. After pregnancy exposure to TiO_2_NPs, the locomotor activity of offspring was evaluated by open field test at postnatal day (PD) 21 and PD49. **A** Effects of TiO_2_NPs on the total distance. **B** Effects of TiO_2_NPs on the central square duration. **C** Effects of TiO_2_NPs on the distance moved in center. **D** The representative track maps in the open field test. Data were presented as mean ± S.E.M. Statistical analysis was performed by using repeated-measure ANOVA or independent student *t* test or Mann–Whitney U test

Open field test is a widely used method for the assessment of locomotor activity in rodent animals [37]. In this study, the locomotor activity was evaluated in the offspring at their age of PD21 and PD49, respectively. The results demonstrated that the total distance, central square duration and distance moved in center were all unchanged in TiO_2_NPs-treated group at time point of PD21 in comparison to vehicle group (Fig. 6A–C). Intriguingly, the performance of offspring in the open field apparatus changed remarkably when they reached the adulthood. As compared with vehicle controls, the TiO_2_NPs-administrated offspring exhibited much lower central square duration, shorter total distance, and shorter distance moved in center at PD49 (Fig. 6A–C). The representative track maps in the open field test were shown in Fig. 6D. These results imply that the inhibition of locomotor activity in offspring induced by pregnancy exposure of TiO_2_NPs did not occur in the adolescent but emerged in the adulthood.

### Pregnancy exposure to TiO_2_NPs impaired the learning and memory ability of offspring at PD49 but not emerged at PD21

**Fig. 7:**
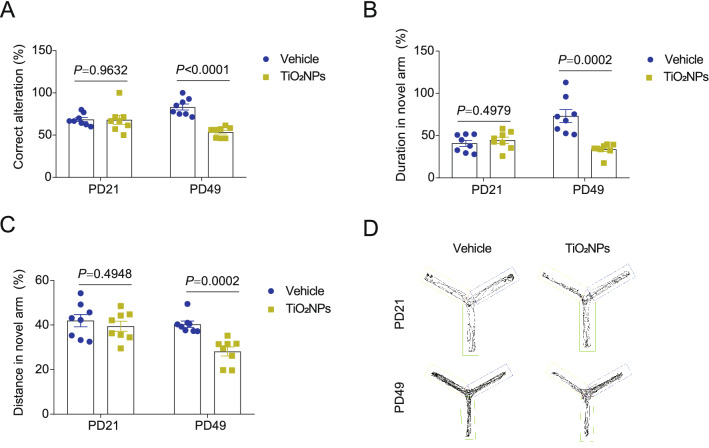
Pregnancy exposure to TiO_2_NPs impaired the learning and memory ability of offspring at PD49 but not emerged at PD21. After pregnancy exposure to TiO_2_NPs, the learning and memory ability of offspring was evaluated by Y maze at postnatal day (PD) 21 and PD49. **A** Effects of TiO_2_NPs on the correct alteration. **B** Effects of TiO_2_NPs on the duration in the novel arm. **C** Effects of TiO_2_NPs on the distance in the novel arm. **D** The representative track maps in the Y maze. Data were presented as mean ± S.E.M. Statistical analysis was performed by using repeated-measure ANOVA or independent student *t* test or Mann–Whitney U test

Y maze was employed to investigate whether pregnancy exposure to TiO_2_NPs affected the learning and memory ability of offspring. As shown in Fig. 7A, the results demonstrated that there was no significant difference on the percentage of correct alteration between two groups at PD21. On the contrary, the percentage of correct alteration was profoundly decreased in TiO_2_NPs-treated animals as compared with those in the vehicle group at PD49. The spontaneous alternation of Y maze, a measure of spatial working memory, was subsequently used to measure the willingness of animals to explore new environments [38]. As expected, similar trends were observed on the duration and distance in the novel arm (Fig. 7B and C). The representative track maps in the Y maze were shown in Fig. 7D. These results together signify that impairment of learning and memory ability in offspring induced by pregnancy exposure of TiO_2_NPs did not occur postnatally at PD21 but emerged in the adulthood.

### Pregnancy exposure to TiO_2_NPs resulted in anxiety-like behavior in offspring at PD49 but not presented at PD21

**Fig. 8:**
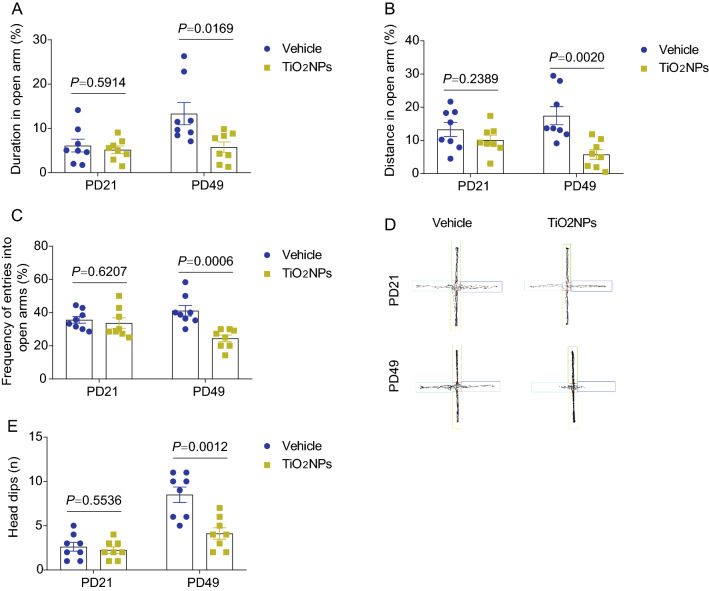
Pregnancy exposure to TiO_2_NPs resulted in anxiety-like behavior in offspring at PD49 but not presented at PD21. After pregnancy exposure to TiO_2_NPs, the anxiety-like behavior in offspring was evaluated by elevated plus maze at postnatal day (PD) 21 and PD49. **A** Effects of TiO_2_NPs on the duration in the open arms. **B** Effects of TiO_2_NPs on the distance in the open arms. **C** Effects of TiO_2_NPs on the frequency of entries into open arms. **D** The representative track maps in the elevated plus maze. **E** Effects of TiO_2_NPs on the head dips. Data were presented as mean ± S.E.M. Statistical analysis was performed by using repeated-measure ANOVA or independent student *t* test or Mann–Whitney U test

To determine if pregnancy exposure to TiO_2_NPs lead to anxiety-like behavior in offspring, the elevated plus maze was conducted. At PD21, the results elucidated that administration of TiO_2_NPs during pregnancy did not alter the duration in the open arms, the distance in the open arms as well as the frequency of entries into open arms in comparison to vehicle controls (Fig. 8A–C). Conversely, these indicators in the elevated plus maze were all declined at PD49 in the TiO_2_NPs-treated group when compared with vehicle group. The representative track maps in the elevated plus maze were shown in Fig. 8D. Moreover, similar trends were observed in the head dips (Fig. 8E). The results together suggest that pregnancy exposure of TiO_2_NPs cause the anxiety-like behavior in offspring that is not presented in adolescent but becomes obviously in adulthood.

### Pregnancy exposure to TiO_2_NPs disturbed compositions of microbial community in offspring at PD49 but not occurred at PD10 and PD21

**Fig. 9:**
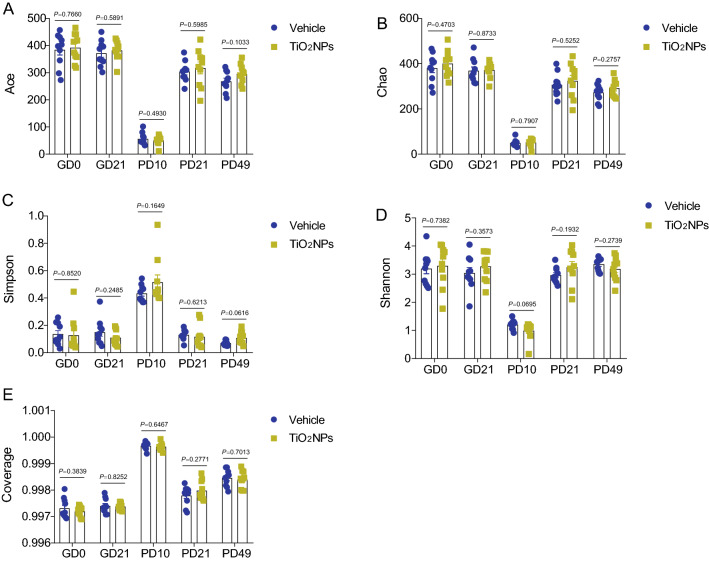
Pregnancy exposure to TiO_2_NPs did not affect the α-diversity indices at PD10, PD21 and PD49. After 16S rRNA sequencing, the richness and evenness of gut microbiota were evaluated by α-diversity indices at postnatal day (PD) 10, 21 and 49. **A** Effects of TiO_2_NPs on the Ace index. **B** Effects of TiO_2_NPs on the Chao index. **C** Effects of TiO_2_NPs on the Simpson index. **D** Effects of TiO_2_NPs on the Shannon index. **E** Effects of TiO_2_NPs on the Coverage index. Data were presented as mean ± S.E.M. Statistical analysis was performed by using repeated-measure ANOVA or independent student *t* test or Mann–Whitney U test

**Fig. 10:**
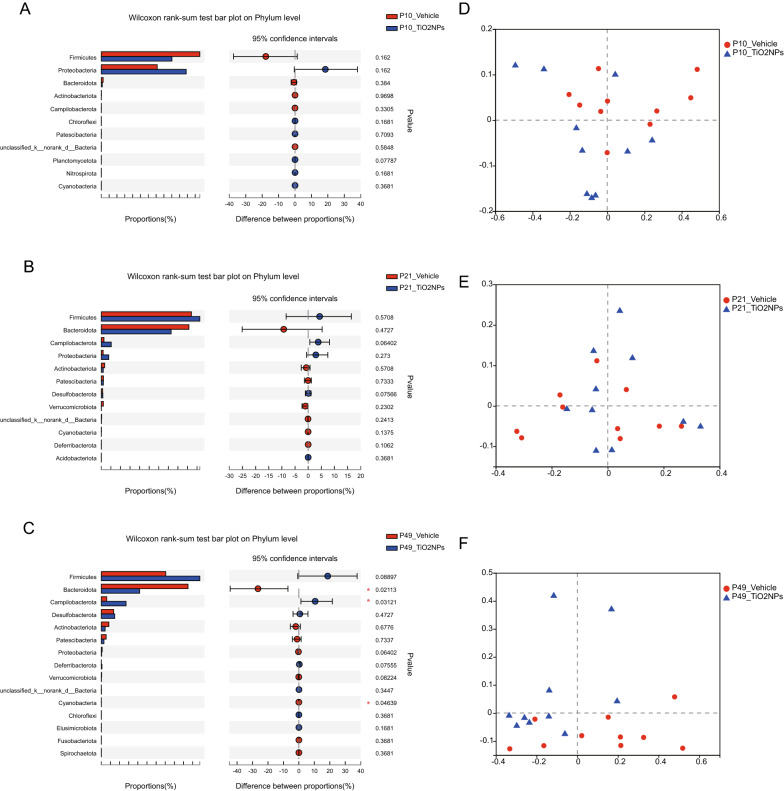
Pregnancy exposure to TiO_2_NPs disturbed compositions of microbial community in offspring at PD49 but not occurred at PD10 and PD21. After 16S rRNA sequencing, compositions of microbial community in offspring were assessed at postnatal day (PD) 10, 21 and 49. **A**–**C** Wilcoxon rank-sum test bar plot on Phylum level at different time points tested. **D**–**F** Principal Co-ordinates Analysis (PCoA) on phylum level

To further investigate the role of intestinal microbiota in TiO_2_NPs-induced neurobehavioral impairments, the 16S rRNA gene sequencing was used to analyze bacterial DNA in feces collected from dams and offspring. The 16S rRNA sequences were clustered at greater than 97% sequence similarity on the OTUs level. In this study, the data did not show any significant differences on the α-diversity indices, including Ace, Chao, Simpson, Shannon, in dams at both GD0 and GD21 (Fig. 9A–D). Similarly, we also did not observe apparent changes on the α-diversity indices in the offspring of TiO_2_NPs-treated animals as compared with those of vehicle controls at PD10, PD21 and PD49 (Fig. 9A–D). Herein, the completeness of the sequencing was evaluated by coverage, all of which were greater than 99%, suggesting that the majority of bacterial species presented in the tested fecal samples of dams and offspring had been detected (Fig. 9E). These results together signify that pregnancy exposure of TiO_2_NPs does not affect the α-diversity in both dams and offspring. No obvious alterations were observed on the Phylum level at PD10 and PD21 between two groups (Fig. 10A and B). Interestingly, the results demonstrated that, as compared with vehicle controls, the relative abundance of *Bacteroidota* and *Cyanobacteria* were significantly declined, whereas the relative abundance of *Campilobacterota* was profoundly enhanced in offspring of TiO_2_NPs-treated mice at PD49 on the Phylum level (Fig. 10C). Similar trends were also observed in the PCoA, which was used for the assessment of β-diversity (Fig. 10D–F). These results together indicate that pregnancy exposure to TiO_2_NPs does not affect the balance of gut microecology in dams. Notably, this treatment disturbs compositions of microbial community in offspring at PD49 but not appears in the young age at PD10 and PD21.

### Pregnancy exposure to TiO_2_NPs caused the obvious pathological damage in intestine and brain of offspring at PD49

**Fig. 11:**
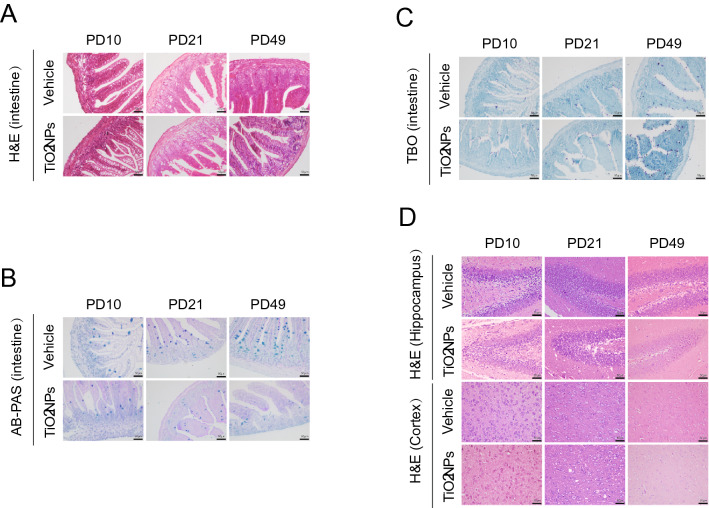
Pregnancy exposure to TiO_2_NPs caused the obvious pathological damage in intestine and brain in offspring at PD49. After indicated treatment, the tissues were collected at postnatal day (PD) 10, 21 and 49. **A** The morphological changes of intestine tissues were observed in H&E staining. **B** AB-PAS staining was used to observed the changes on the number and size of goblet cells. **C** Alterations on the number of mast cells were observed in TBO staining. **D** The morphological alterations in the hippocampus and cerebral cortex tissues were observed in H&E staining. Scale bar, 50 μm

The tissue integrity of intestine in offspring was firstly evaluated by histopathological analyses at PD10, PD21 and PD49, respectively. In the H&E staining assay, the results demonstrated that pregnancy exposure to TiO_2_NPs caused the irregularly shaped or completely destroyed villi in the intestine of offspring, which became obviously at PD 49 (Fig. 11A). Similarly, the obtained results in AB-PAS staining assay demonstrated that the number and size of goblet cells were significantly reduced in the offspring of TiO_2_NPs-treated animals at PD 49 (Fig. 11B). In contrast, the results in toluidine blue staining assay illustrated that the number of mast cells were apparently increased in the offspring of TiO_2_NPs-treated animals at PD 49 (Fig. 11C). Given both hippocampus and cerebral cortex are the major brain regions that involved in the regulation of gut-brain bidirectional communication, the potential pathological damage of these two regions were evaluated by H&E staining assay at PD10, PD21 and PD49, respectively. As shown in Fig. 11D, the results illustrated that pregnancy exposure to TiO_2_NPs did not cause apparent tissue injuries in the hippocampus of offspring. On the contrary, the shrink nuclear and deformed cell body, and the decreased number of neurons were appeared in the cerebral cortex area of offspring in TiO_2_NPs-exposed group. Moreover, these pathological changes were much more obvious at PD49. These findings indicate that pregnancy exposure to TiO_2_NPs results in the pathological damage in intestine and cortex tissues, and becomes obviously at adulthood of offspring.

### Pregnancy exposure to TiO_2_NPs specifically disrupted the gut-brain axis in offspring

**Fig. 12:**
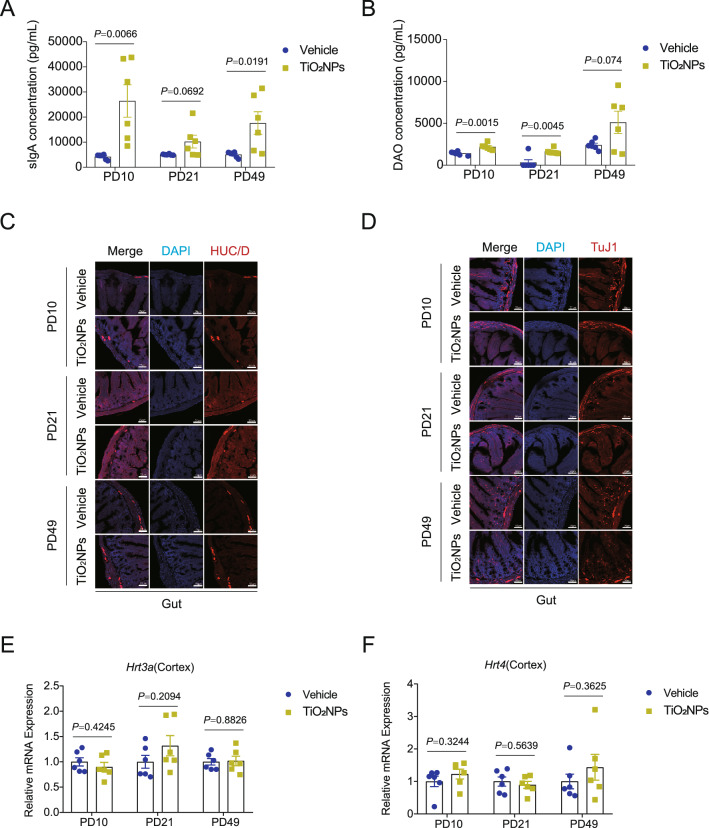
Pregnancy exposure to TiO_2_NPs caused the disruption of gut-brain associated immune response but did not affect the levels of enteric neuron and gut-derived neurotransmitters and peptides in offspring. After designed treatment, the indicators were assessed at postnatal day (PD) 10, 21 and 49, respectively. **A**-**B** Effects of TiO_2_NPs on sIgA and DAO contents in gut were determined by using ELISA assay. The HuC/D and TuJ1 expressions were determined by immunofluorescence staining, and the representative images were shown in (**C**) and (**D**). Scale bar, 50 μm. **E**–**F** Effects of TiO_2_NPs on the *Hrt3a* and *Hrt4* mRNA expressions in the cortex. Data were presented as mean ± S.E.M. Statistical analysis was performed by using repeated-measure ANOVA or independent student *t* test or Mann–Whitney U test

**Fig. 13:**
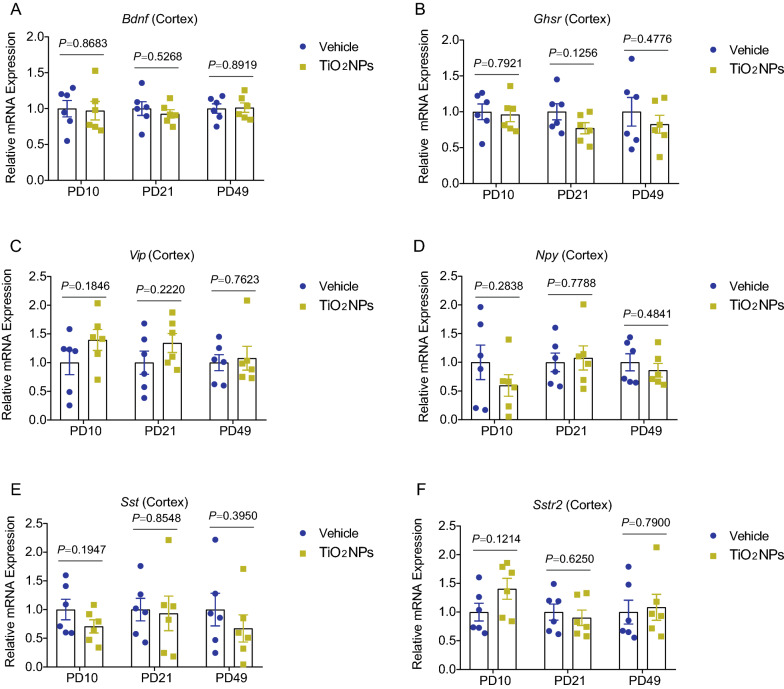
Pregnancy exposure to TiO_2_NPs did not affect the mRNA expressions of gut-brain peptides in offspring. Effects of TiO_2_NPs on the *Bdnf* (**A**), *Ghsr* (**B**), *Vip* (**C**), *Npy* (**D**), *Sst* (**E**) and *Sstr2* (**F**) mRNA expressions in the cerebral cortex tissues at postnatal day 10, 21 and 49. Data were presented as mean ± S.E.M. Statistical analysis was performed by using repeated-measure ANOVA or independent student *t* test or Mann–Whitney U test

Gut-brain axis is a complex bidirectional communication network existing within gut and brain. The gut-brain crosstalk is mainly mediated by immunological, neural signals and hormonal pathways. To further investigate whether pregnancy exposure of TiO_2_NPs induces neurobehavioral impairments through the gut-brain axis, the major indicators involved in gut-brain crosstalk were all detected in this study. At first, the enteric immune response of the offspring was assessed by determination of intestinal contents of sIgA and DAO, both of which were participated in the regulation of intestinal immune barrier function. As shown in Fig. 12A and B, the results demonstrated that, after pregnancy exposure to TiO_2_NPs, the contents of sIgA and DAO were significantly enhanced as compared with vehicle controls, indicating that immunological pathway was activated in the offspring of TiO_2_NPs-exposed animals. Next, to evaluate the state of enteric neurons within the intestine, the expression of neuronal makers HuC/D and TuJ1 were determined by immunofluorescence assay. The results showed that there were no apparent alterations on the expression of HuC/D and TuJ1 in the intestine of offspring between two groups at PD10, PD21 and PD49 (Fig. 12C and D). These data imply that neural signals may not involve in the adverse neurodevelopmental effects of TiO_2_NPs. Lastly, the mRNA expressions of major gut-derived neurotransmitters and gut-brain peptides were determined. As shown in Fig. 12E and F, the results clearly illustrated that the gut-derived neurotransmitters, *Hrt3a* and *Hrt4*, did not change obviously in offspring of TiO_2_NPs-treated group in comparison to vehicle group at three tested time points. Moreover, No significant differences were observed on the mRNA expressions of gut-brain peptides including *Bdnf*, *Ghsr*, *Vip*, *Npy*, *Sst* and *Sstr2* between two groups at PD10, PD21 and PD49 (Fig. 13A–F). These results together suggest that gut-derived neurotransmitters and gut-brain peptides may not participate in the regulation of TiO_2_NPs-induced neurobehavioral impairments.

## Discussion

Exposure to TiO_2_NPs, either intentional or unintentional, is the most prevalent exposure scenario in the daily life. Humans, including adult, elderly, pregnant women and children, are all very frequently exposed to TiO_2_NPs-contained foods or commercial products [1–3]. Recently, the toxicity of TiO_2_NPs on different organs were widely reported, although they were thought to be low toxic in the past decades [5, 9]. Notably, the evidence of neurodevelopmental toxicity caused by TiO_2_NPs in mammals is still limited. Thus, in this study, by administration of pregnant C57BL/6 J mice at dose of 150 mg/kg from GD8 to GD21, the potential effects of TiO_2_NPs on the neurodevelopment were systemic evaluated. Herein, we demonstrated the intriguing findings for the first time that exposure of TiO_2_NPs during pregnancy resulted in the neurobehavioral impairments, including locomotor inhibition, learning and memory deficits and anxiety-like behavior, that were not significant postnatally but emerge in adulthood of offspring. Similarly, the results also elucidated that the delayed appearance of intestinal dysbiosis in TiO_2_NPs-exposed offspring was highly associated with neurobehavioral impairments, which might occur through disruption of gut-brain axis in the intestine.

The developmental toxicity of TiO_2_NPs has been reported in the previous studies conducted mainly by zebrafish, mice and rats [15]. By using pregnant Sprague–Dawley rats, the increased levels of titanium in the maternal liver, brain and placenta were observed after orally administered of rats with TiO_2_NPs from GD 6 to GD19 at dosage levels of 0, 100, 300 and 1000 mg/kg/day during pregnancy [39]. Similar results were also obtained in the CD-1 mice, showing that treatment of TiO_2_NPs from GD0 to GD17 increased the titanium concentrations in maternal serum, placenta and fetus. Moreover, TiO_2_NPs can delay the normal development and induce skeletal malformation in offspring after crossing the blood-fetal barrier and placental barrier [40]. There is no available evidence now showing that pregnancy exposed TiO_2_NPs in mother mice may appear in the intestine of their offspring. However, it is well known that orally administered TiO_2_NPs during pregnancy significantly increased the titanium concentrations in fetal serum, implying that TiO_2_NPs may transport into major organs (including intestine) of body via blood systemic circulation.

It is noteworthy that, TiO_2_NPs were able to found in the fetal brain, indicating that potential neurodevelopment toxicity in the offspring [41]. Indeed, prenatal exposure of TiO_2_NPs was shown to induce genital and cranial nerve system injuries and affect the neurogenesis in the hippocampus of offspring [42, 43]. On the contrary, some investigations revealed the opposite effects. For instance, Warheit et al. elucidated that pregnancy exposure to different sizes of TiO_2_NPs daily from GD6 to GD20 did not trigger maternal and developmental toxicity at any dose level tested [44]. Results from Lee et al., confirmed that prenatal exposure to TiO_2_NPs by oral gavage did not induce marked toxicities in maternal animals or embryo-fetal development endpoints [39]. In this study, we also did not observe any significant alterations on the parameters of neonatal sensory and motor development, developmental milestones and the neurobehaviors postnatally in the offspring. Intriguingly, the impairments on locomotor activity, learning and memory ability and anxiety-like behaviors emerged when the offspring reached adulthood. The discrepancies on the developmental consequences induced by TiO_2_NPs are considered to be caused by differences in routes of exposure, duration of exposure, physicochemical characteristics of nanoparticles etc. Notably, the delayed appearance of neurobehavioral impairments strongly indicates that TiO_2_NPs are not completely safe for the pregnant women. Despite we did not observe the obvious changes on the maternal behaviors after pregnancy exposure of TiO_2_NPs, it did not mean administration of TiO_2_NPs produced no overt toxicity in the pregnant mice themselves. Our results also indicated that pregnancy exposure to TiO_2_NPs was able to cause the persistent adverse effects in pregnant mice themselves (data not shown). Moreover, exposure of TiO_2_NPs is able to induce infertility phenotype in the animals [45, 46]. Therefore, further investigations are seriously required to re-evaluate the toxicity of TiO_2_NPs.

Delayed neurobehavioral impairment is commonly defined as a delayed onset of neurotoxic effects on behaviors resulting from a single or repeated exposure to environmental xenobiotics [47]. The delayed neurotoxic effects have been reported in some chemicals, such as organophosphates [48] and PCB126 [49], with the absence of symptoms associated with acute toxicity. These delayed toxic effects of TiO_2_NPs have not been reported at present, although the neurobehavioral impairments induced by TiO_2_NPs have been extensively studied. In the previous study of our lab, we had already revealed that oral exposure to TiO_2_NPs for 30 days was capable of inducing the inhibition of locomotor activity in adult mice, but this treatment did not cause any significant impact on the function of spatial learning and memory [32]. Likewise, administered to animals with TiO_2_NPs at 20 mg/kg for 20 days by intraperitoneally injection was shown to increase the anxious index significantly as compared with controls [50]. Pregnancy exposed TiO_2_NPs at 6.5 nm particle size is able to deposit in the brain of offspring, and thereby causing the retarded axonal and dendritic outgrowth through excessive activation of the mitogen-activated protein kinase signaling pathway [51]. Notably, in the study conducted by Cui et al., prenatal exposure to TiO_2_NPs produced the significant oxidative damage to nucleic acids and lipids in the brain of newborn offspring, and thus, resulting in the depressive-like behaviors during adulthood. These findings imply that environmental stress induced by TiO_2_NPs during fetal life may be implicated in neurobehavioral impairments in adulthood [52]. This phenomenon is similar with the results observed in this study showing the delayed emergence of neurotoxic effects induced by TiO_2_NPs.

The detailed mechanisms underlying how TiO_2_NPs caused the delayed neuro behavioral impairments remain largely unknown. Herein, there are two potential explanations: firstly, the ingested TiO_2_NPs during pregnancy may cross the placenta barrier and deposit in the different organs of fetus, the persistent retention of TiO_2_NPs in the offspring mice may slightly affect the brain function at early stage, and this adverse effect is possibly amplified over time, therefore leading to the delayed impairments in neurobehaviors. Secondly, the learning and memory, locomotor and anxiety-like behavior tested in this study are all belonging to the higher neuronal activity, which is also the highest level of integrative function of brain. Thus, at the early age of animal, these brain functions are not fully developed. The damage in these complex neurobehaviors may not appear obvious in the young brain but become severe as time goes by.

Recently, increasing evidence shows the existence of a bidirectional communication between gut microbiota and brain [53, 54]. Importantly, the microbiota with in the gut can greatly affect many neurobehaviors, including learning and memory, sensory perception, motor functions etc. [53]. Previous studies also proposed that gut microbiota influences the neurobehaviors mainly by utilizing endocrine, neuroendocrine and immunologic signaling pathways [55, 56]. Moreover, microbiota state in the gut is highly associated with the fetal brain development and functions [57, 58]. Therefore, disruption of gut-brain axis function induced by environmental stress is considered to be contributed to the impairments of neurobehaviors. In this study, after pregnancy exposure of animals with TiO_2_NPs, the results revealed that no significant changes were obtained in α-diversity in dams and their offspring between TiO_2_NPs-treated group and vehicle group. Intriguingly, herein, the delayed adverse effects on the gut microbiota were observed in offspring of TiO_2_NPs-treated mice at PD49, showing the decreased abundance of *Bacteroidota* and *Cyanobacteria* and increased abundance of *Campilobacterota* on the Phylum level. Moreover, no apparent alterations on the microbial communities were obtained at PD10 and PD21 between two groups. The possible explanation for this phenomenon is that the enduring shifts in the microbiota composition may be presented by perturbation of TiO_2_NPs at a timepoint because of the microbiota within the gut may not fully matured or is generally unstable during early life after delivery [59]. Coincidentally, this delayed impact of TiO_2_NPs on the gut microbiota is highly linked with the neurobehavioral impairments of offspring in adulthood. Therefore, the toxicity on the gut microbiota may be regarded as a potential mechanism by which TiO_2_NPs lead to neurobehavioral impairments. It is of necessity to reconsider the health risk of prenatal exposure of TiO_2_NPs in the context of their contribution to intestinal dysbiosis in offspring.

The impact of TiO_2_NPs on the intestinal microbiota can be direct or indirect. At first, orally administrated TiO_2_NPs in dams may directly deposit in offspring when passing through the placental barrier [39, 41]. They are able to affect the microbiota of offspring persistently after delivery and induce an unbalanced gut bacterial ecosystem in adulthood. The dysbiosis induced by TiO_2_NPs may interrupt specific set of metabolic pathway or genome possessed by different bacterial species [23, 32, 60]. Additionally, TiO_2_NPs may indirectly impact the intestinal microbiota through the influence on physiological functions of host. In this study, the data showed that the contents of sIgA and DAO were remarkably elevated in offspring of TiO_2_NPs-treated animals at different time point tested, indicating that immunological pathway was activated in the offspring of TiO_2_NPs-exposed animals. However, no apparent alterations were observed on other indicators, such as enteric neurons, gut-brain peptides and neurotransmitters. These findings further suggest the delayed neurobehavioral impairments induced by TiO_2_NPs possibly occur through the activation of specific immunological pathway between gut and brain. In this study, the results also revealed that oral administration of TiO_2_NPs in dams at 150 mg/kg did not cause the intestinal and brain injuries in offspring at PD10 and PD21 but became apparent in adulthood. There would be two possible reasons for this interest phenomenon. Firstly, the intestinal damage induced by TiO_2_NPs may leave offspring vulnerable to excess harmful gut bacteria, increased risk of adverse effects in gut, and ongoing neurobehavioral impairments through gut-brain axis. Secondly, the ingested TiO_2_NPs by dams may deposit in the different organs of offspring, including brain [41]. The accumulated TiO_2_NPs in the brain of offspring may do not cause the tissue injury at early life after delivery, but the chronic continuous stimulation of nanoparticles possibly results in tissue damage over time.

There are several limitations in this study. First, the experimental design did not include more than two doses of TiO_2_NPs based on the consideration of Replacement, Reduction and Refinement principles in toxicological studies, especially for the prenatal animal model. Second, there exist significant differences between mice and humans, so the dosage used in this study was not completely equal to acceptable daily intake of TiO_2_NPs or the concentrations that presented in the natural environment. Third, it is hardly to verify the relationship definitely between “cause”, the specific intestinal microbiota, and “effect”, the neurobehavioral impairments due to most of the microbiota is not available isolates or hard to cultivate.

In summary, the results herein demonstrated for the first time that oral exposure of TiO_2_NPs during pregnancy did not affect the maternal behaviors and offspring postnatal development at the early life after birth. However, this administration of TiO_2_NPs profoundly led to neurobehavioral impairments and brain tissue injuries in the offspring when they reached adulthood. We also proposed a novel insight that the intestinal dysbiosis might be highly associated with the delayed appearance of neurobehavioral impairments in offspring induced by TiO_2_NPs. These findings will provide a new clue for better understanding the neurodevelopmental toxicity of TiO_2_NPs, and call for comprehensive health risk assessment of TiO_2_NPs on the susceptible population, such as pregnant women.

## Supplementary Information

**Additional file 1: Table S1.** Primer sequences of target genes.

## Data Availability

All the original data are available upon reasonable request for correspondence authors.
