# Supplementary material for: Pregnancy exposure of titanium dioxide nanoparticles causes intestinal dysbiosis and neurobehavioral impairments that are not significant postnatally but emerge in adulthood of offspring
Source: J Nanobiotechnology. 2021 Aug 6;19:234. doi: 10.1186/s12951-021-00967-5 (PMC8349049; doi:10.1186/s12951-021-00967-5)
Supplement: Supplementary file 1 — Additional file 1: Table S1. Primer sequences of target genes. [file 12951_2021_967_MOESM1_ESM.doc]

Table. S1 Primer sequences of target genes

| Gene | Forward (5’-3’) | Reverse (5’-3’) | Length (bp) |
| --- | --- | --- | --- |
| *Bdnf* | *CTCCTCTACTCTTTCTGC* | *CACTCGCTAATACTGTCA* | 130 |
| *Ghsr* | *AGAGAAAGGAATCCAAGAAG* | *CATGCTGCTGATACTGAG* | 174 |
| *Hrt3a* | *AACAAGACTGATGACTGCTCAG* | *GATGGAGGATAGCTCTTGCAAG* | 159 |
| *Hrt4* | *AGGTCCGTGGAGAAGGTCGTG* | *CACAGCCACCATCACCAGCAG* | 90 |
| *Vip* | *GTAGTGAGTAGGCTGGAT* | *TGGCATTTCTTGACACAT* | 142 |
| *npy* | *CATGGCCAGATACTACTC* | *CCTTCATTAAGAGGTCTGA* | 110 |
| *Sst* | *CCAACCAGACAGAGAATG* | *ACAGGATGTGAATGTCTTC* | 170 |
| *Sstr2* | *CAACATCTACATCCTTAACCT* | *GATACTGGTGAACTGATTGA* | 157 |
| *β-actin* | *CGTTGACATCCGTAAAGA* | *CAGAGCAGTAATCTCCTTC* | 103 |
